# *Papás Activos*: Associations between Physical Activity, Sedentary Behavior and Personal Networks among Fathers Living in Texas *Colonias*

**DOI:** 10.3390/ijerph17249243

**Published:** 2020-12-10

**Authors:** Tyler Prochnow, M. Renée Umstattd Meyer, Megan S. Patterson, Megan E. McClendon, Luis Gómez, Stewart G. Trost, Joseph Sharkey

**Affiliations:** 1 Department of Public Health, Robbins College of Health and Human Sciences, Baylor University, One Bear Place #97303, Waco, TX 76798, USA; renee_umstattd@baylor.edu; 2 Department of Health and Kinesiology, College of Education and Human Development, Texas A&M University, 4243 TAMU, College Station, TX 77845, USA; megpatterson@tamu.edu; 3 Department of Health & Human Performance, Texas State University, 601 University Dr, San Marcos, TX 78666, USA; mem473@txstate.edu; 4 Department of Health Promotion & Community Health Sciences, School of Public Health, Texas A&M University, 212 Adriance Lab Rd. 1266 TAMU, College Station, TX 77843, USA; gomez@tamu.edu (L.G.); jrsharkey@tamu.edu (J.S.); 5 School of Exercise and Nutrition Sciences, Institute of Health and Biomedical Innovation, Queensland University of Technology, 2 George St, Brisbane City, QLD 4000, Australia; s.trost@qut.edu.au

**Keywords:** social network analysis, Mexican-heritage, family systems

## Abstract

Despite growing health disparities in Latino populations related to lack of physical activity (PA), little is known regarding the impact of social networks on PA and sedentary behavior among a sample of Latino fathers residing in functionally rural *colonias*. Fathers wore accelerometers and responded to questions regarding their self-efficacy and characteristics of who they were active with most often. Fathers (*n* = 47) attained a mean of 73.3 min of moderate-to-vigorous PA (SD = 23.8) per day and were sedentary for a mean of 364.0 min (SD = 74.4) per day. In total, fathers reported 205 alters and significantly more family members (M = 3.60, SD = 1.64) than friends (M = 0.77, SD = 1.37). Sedentary time was positively associated with number of peers and inversely related to the number of children reported. Minutes of moderate-to-vigorous PA was significantly associated with greater self-efficacy and number of family members reported. This study contributes to the evidence by further examining PA correlates of Latino fathers from functionally rural *colonia* communities. Additionally, this study supported both family systems theory and the socio–ecological model as it details the interpersonal and familial influences of PA behavior. Thus, supports for family activity promotion and programs which impact familial norms and activity at the family level may be particularly useful.

## 1. Introduction

The promotion of physical activity (PA) is a large public health pursuit due to improved physical and mental functioning, and decreased risk of chronic diseases [1]. Adults are recommended to be physically active in moderate to vigorous intensity for more than 150 min each week, in addition to engaging in muscle strengthening exercises on at least two days [2]. Adults are suggested to engage in activity up to 300 min each week for additional health benefits [2]. Unfortunately, the rate of adults meeting these recommendations is declining in the United States and Latin America [3]. Additionally, adults in rural communities are less likely to meet PA guidelines [4]. Many researchers credit sedentary lifestyles and other life obligations as potential reasons for this decline [5]. Beyond this overall decline, there are further racial and ethnic disparities in PA attainment [6]. More specifically, Latinx male adults in the United States report significantly less PA than other racial and ethnic groups [7]. This is more concerning when considering Latinx adults are disproportionately affected by obesity and obesity-related chronic disease such as diabetes and heart disease [8,9]. Because PA lowers the risk of both diabetes and heart disease [1], promoting PA in this population is increasingly vital.

One important yet often systematically underserved Latinx population resides in the *colonias* on the U.S.–Mexico border. These *colonias* are defined as economically distressed communities consisting of low or very-low income households based on the Federal poverty index located at or near the U.S.–Mexico border area with an outer range stretching from 50–150 miles into the U.S. [10]. In Texas alone, it is estimated that over 860 *colonias* provide homes for nearly 150,000 residents [10]. Rurality is just one characteristic of this systematically underserved population, as *colonias* face multidimensional challenges related to their rural geographic location, increased rates of poverty, Mexican-heritage populations, and disproportionate obesity rates often due to decreased PA and increased sedentary behavior [11]. Furthermore, the U.S. Department of Health and Human Services has deemed this portion of the U.S.–Mexico border region a medically underserved area due to the social and health barriers, rates of poverty and chronic disease present in these communities [12]. Approximately 500,000 people live in *colonias* located along the Texas–Mexico border [10]. Specifically, adults in this area have rates of obesity (55.5%) and diabetes (32.5%) [13] much higher than the national averages (29.9% and 10.5%, respectively) [14,15]. Similarly, these *colonias* may typify health disparities and struggles within other new destination immigrant communities throughout the U.S. which may struggle to adequately address the health needs of a changing demographic [16]. Hence, understanding the specific influences on health and PA within this unique population is needed. Contextually specific studies are then needed in order to address these specific influences and disparities in order to promote health within these communities.

While mothers are often considered the family gatekeepers within Latinx family contexts and primarily responsible for caregiving and health [17,18], recently researchers have recognized the impact of Latinx fathers on the health and wellbeing of their children [19,20]. Freeman et al. [21] called for a renewed focus on the impact of fathers within child obesity research. Studies suggest fathers’ PA is not only beneficial for their own health, but potentially beneficial for their children by modeling healthy behaviors [22,23]. Family systems theory posits that a family functions as a system wherein people are expected to interact with and respond to one another in certain ways, creating family norms and social influence on health behavior [24]. Similarly, the socio–ecological model encourages researchers to consider complex health issues from multiple vantage points, including examining interpersonal effects on health behavior [25]. The social environment influences PA behaviors through social support, perceived norms, and co-participation [26]. Further, both family systems theory and the socio-ecological model call for a further understanding of impact of relationships and social influences on PA [24,27]. However, there is presently a lack of research findings regarding social influences on Latinx fathers despite the health disparities and theorized impact on child health. Furthermore, a more substantial understanding of the social influences related to father PA and sedentary behavior would provide a more complete view of the dynamics of the family systems theory and interpersonal level of the socio–ecological model within this systematically underserved population.

Social network analysis (SNA) can be used to assess and quantify this social influence from connections [28]. SNA allows researchers to understand the social structure and influences affecting a person and their health behaviors [29]. More specifically, egocentric, or personal network analysis, is focused on examining someone’s immediate, local network. Egocentric network analysis is accomplished by documenting the close social connections of each individual being surveyed (also called ego) [29]. Egocentric network studies sample individuals and ask them for their most salient connections or relationships (these people are called alters). In this manner traditional sampling techniques can be used and anonymity can be maintained as real names or identities of the people in the network (alters) are not necessary [30]. Using SNA within this culturally and contextually specific population would further add to the literature on social influences among this group.

Researchers have found many significant differences in the way different racial and ethnic groups form, maintain, and interact with their social networks [31]. These differences may be important in understanding the role of social networks on PA. For example, Latinx families exhibit higher levels of familism and collectivism when compared to non-Latinx White families [32]. Familism is an emphasis on family relationships or having loyalty and pride in one’s family [33,34]. Similarly, collectivism is defined as providing financial or social support to one’s familial unit above all else [35]. In a study investigating the impact of social connections on Mexican-heritage adults, only 58% reported at least one member of their network who encouraged them to engage in regular PA; meanwhile only 10.8% of total network members were said to be encouraging [36]. Furthermore, having at least one social network member who encouraged participants to engage in regular PA was associated with participants’ motivation to engage in regular PA [36]. However, there is still a dearth of research detailing the role of a father’s social network on his objectively measured PA behaviors.

### Specific Aims

Therefore, this paper aims to investigate social network influences attributing to the PA and sedentary behavior of fathers living in Texas *colonias* by using egocentric social network analysis. This analysis will fill a gap in the literature concerning Latinx, specifically Mexican-heritage *colonia* residents, fathers and culturally specific social networks within a functionally rural community. The analysis presented here represents preliminary baseline pilot data with vital specificity to the *colonais.* Specificity, in this case, adds to the literature regarding the social network influence on paternal PA which has widely been called for in previous studies [21,22,23] yet has not occurred in these culturally and contextually specific communities. Learning more about how social networks impact PA behaviors among this sample could provide information on how to leverage social contacts to promote healthy behaviors within these *colonias*.

## 2. Materials and Methods

### 2.1. Setting

This study is part of the larger Salud Para Usted y Su Familia (SPUSF) (Health for You and Your Family) parent project to develop, implement, and evaluate a father-focused, family-centered, intervention program based on active living, healthy eating, and family communication. This program was culturally tailored for Mexican-heritage families living in functionally rural *colonia* areas along the South Texas border with Mexico.

This project benefited from the use of a *promotora* research model, *Promotora*-researchers are members of the community who are trained in public health, community outreach, and research techniques [37]. This model is particularly effective in developing culturally relevant research and vital to building rapport with traditionally hard to reach populations [38]. *Promotora*-researchers actively participated in every step of the survey development process, including drafting and translating the survey as well as administering them to all father participants.

Participants of the program were recruited from four randomly identified geographic clusters located near unincorporated San Carlos, Texas. *Promotora*-researchers completed door-to-door screening and re-contacted participants from previous studies whom had provided consent for re-contact in future studies in order to recruit for this study. In total, 308 families were screened and 60 families were recruited into the program. These families were then split into five groups of 12 families. The fifth group (*n* = 13) was not included in this study due to incomplete data. This sample represents a convenience sample for preliminary baseline data analysis. Families needed to meet each of the following criteria to be included: (1) have no food allergies, (2) have no PA restrictions, (3) both parents being at least 21 years of age, (4) have at least one member of the nuclear family, or one directly related family member (i.e., grandparents or great-grandparents to child participant), born in Mexico (to establish Mexican-heritage), (5) have a child 9–11 years old living in the household, (6) both parents preferring to speak, write, and read in Spanish, and (7) both parents actively living in the same household. Fathers from each participating family were recruited to participate in this preliminary study.

### 2.2. Measures

Surveys were administered by researchers in Spanish primarily in the father’s household and answer grids were provided to assist participants with the administration process. In several cases, and to provide the necessary schedule flexibility, surveys were also administered at the participant’s job site, church, or at the home of another family member. Surveys were completed between June 2019 and November 2019. Fathers reported their age as demographic information.

#### 2.2.1. Physical Activity Self-Efficacy

Self-efficacy is a measure of confidence in one’s ability to perform a specific task and is consistently associated with the adoption and maintenance of PA habits [39,40]. PA self-efficacy was measured using a modified version of the exercise self-efficacy scale [41]. In its original form, the scale contains 18-items with each item being measured on a 0–100 scale. The scale wording and response options were modified with feedback from the *promotora*-researchers to better fit the sample and context. Modifications were made to ensure proper translation of meaning and to fit reading and comprehension levels. Research has suggested scales with three simple response options achieve more reliable and satisfactory psychometric performance among nonreaders or those with lower literacy levels [42]. The scale included 14-items such as, “How confident are you that you can be physically active for 30 min at least 5 times a week?”, “How confident are you that you can be physically active when you are feeling tired?”, and “How confident are you that you can be physically active when you are feeling under pressure to get things done?” Response options were reduced to a 3-point scale “unconfident”, “somewhat confident”, and “very confident”. Responses were summed to develop a total scale score. This scale showed acceptable internal reliability (α = 0.67) in this sample [43]. Past studies have shown acceptable results culturally tailoring this scale to Korean [44] and Australian [45] adults.

A confirmatory factor analysis was conducted to examine the modified scale. The one factor, three subfactor structure utilized by Shin, Jang and Pender [44] was transposed on the current scale. The three subfactors were situational, intrapersonal, and internal feelings [44]. Table 1 provides the scale items, item means, and subfactor loadings. Each subfactor had acceptable internal reliability as well (α = 0.78, 0.94, 0.70, respectively) [43].

#### 2.2.2. Social Network Data

Fathers were asked to report the initials or pseudonym of up to five individuals (alters) with whom they were “physically active with most often or actively played with most often” in the previous month. Fathers were limited to five alters as this has been used in past studies to elicit the most salient connections as well as to lessen respondent burden [46,47]. Fathers were then asked questions (name interpreters) to better understand the people in their networks. Fathers reported the sex of each alter, his relationship (spouse, program child, friend, other child, or other) to each alter, whether the alter was physically active regularly (yes, no, or I do not know), and if he thought the alter helped him to be physically active (yes or no).

**Figure 1:**
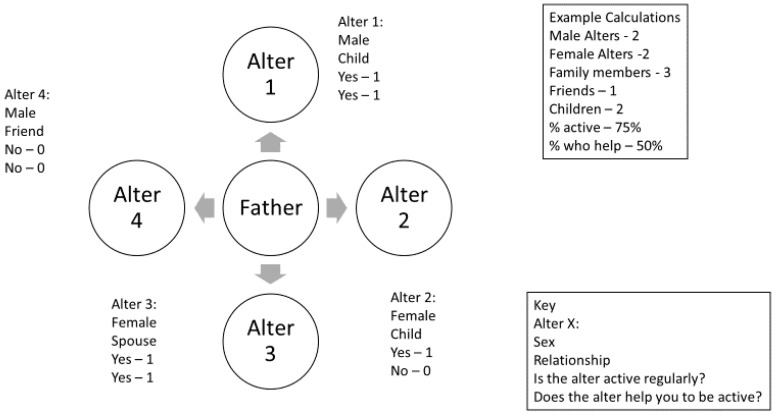
Example father network and calculations.

Network composition variables were calculated for each name interpreter question. For example, the total amount of alters with whom the father reported he was active regularly or the number of children within his network. The relationship name interpreter question was combined to create composition variables such as total family members, total friends, and number of children listed. Combination or aggregation of each variable was done for perception of PA, relation type, and whether the alter helped the father as well. Figure 1 displays a sample father network and example network composition variables calculated.

#### 2.2.3. Objective Physical Activity Measures

Actigraph GT9X accelerometers were used to track minutes of Moderate-to-Vigorous Physical Activity (MVPA) and sedentary minutes per day. Fathers were asked to wear the monitor on their non-dominant wrist 24 h per day for 7 consecutive days. Accelerometers were placed by trained *promotora*-researchers to ensure accurate placement. Raw accelerometer data collected at 30 Hz were processed into PA metrics using a machine-learned random forest classifier specifically designed and validated for assessing PA in free living adults [48]. This activity recognition algorithm uses features in the raw acceleration signal to classify each 10 s window (epoch) as sedentary (lying or sitting still), stationary plus (active sitting, standing still, active standing), walking, or running. Predictions for each 10 s period were then smoothed using a modal filter spanning two lead and two lag epochs. In the current study, MVPA was defined as the sum of daily time spent walking and running. Minutes of MVPA and sedentary minutes were then averaged across all valid wear days to produce a mean MVPA per day and mean sedentary minutes per day for each father.

### 2.3. Data Analysis

Means, standard deviations, and percentages were calculated for demographic variables as well as sedentary minutes and minutes of MVPA per day. Network composition variables were calculated for perception of PA, relation type, whether the alter helped the father, and concurrent activity. Paired sample t-tests were used to determine significant differences in network composition related to alter sex and relationship. Initial correlation matrices were calculated to show bivariate associations between MVPA, sedentary behavior, and network composition variables. Linear regression modeling assessed associations between network variables and sedentary minutes and minutes of MVPA per day. All means, standard deviations, percentages, network composition variables, and regression models were calculated using SPSS v. 25 (IBM, Chicago, IL, USA) [49].

## 3. Results

Fathers in this study (*n* = 47) were on average 38.98 years old (SD = 8.66). Fathers reported a mean self-efficacy score of 19.91 (SD = 5.02), indicating they were somewhat to very confident in their ability to be physically active, on average. Fathers in this sample were highly active; machine learning algorithms estimated fathers attained a mean of 73.3 min of MVPA (SD = 23.8) per day. Additionally, it was estimated fathers were sedentary for a mean of 364.0 min (SD = 74.4) per day.

In total, fathers reported 205 alters. Fathers’ networks consisted of a mean of 4.36 (SD = 1.15) network alters per father. By sex, fathers reported significantly more male alters (M = 2.81, SD = 1.60) than female alters (M = 1.57, SD = 1.44; t(46) = 3.01, *p* < 0.01). Likewise, fathers reported significantly more family members (M = 3.60, SD = 1.64) than friends (M = 0.77, SD = 1.37; t(46) = 6.95, *p* < 0.01). Within those family members, fathers reported a mean of 2.09 children (SD = 1.65); of which 1.17 were male (SD = 1.07) and 0.91 were female (SD = 1.12), on average. There were no significant differences in the mean number of male and female children reported (t(46) = 1.22, *p* = 0.23). On average, fathers reported 4.00 of their network members (SD = 1.18) helped them be physically active. This means roughly 91% of alters were reported to help the fathers be active. Fathers also reported a mean of 3.74 alters who were active regularly (SD = 1.28) accounting for 86% of all alters nominated. Table 2 provides further descriptive analysis of father networks.

Significant bivariate correlations were found positive associations between the number of family members (r(45)= 0.29; *p* = 0.04), self-efficacy(r(45)= 0.29; *p* = 0.04), and minutes of MVPA. Likewise, there was a significant positive association between the number of friends reported and minutes of sedentary activity; r(45) = 0.30; *p* = 0.04. The number of children reported was negatively associated with minutes of sedentary activity; r(45) = −0.31; *p* = 0.03. Table 3 presents the correlation between the covariates measured in this study and minutes of MVPA and sedentary activity.

Significant linear regression models were calculated for mean minutes of sedentary activity (F(2,44) = 4.68, *p* = 0.006, R^2^ = 0.19) and MVPA (F(3,43) = 4.74, *p* = 0.003, R^2^ = 0.25) per day. A father’s minutes of sedentary activity per day were significantly associated with a greater number of friends in his network (β = 47.14, *p* = 0.05) and inversely related to the number of children reported in his network (β = −55.86, *p* = 0.01). A father’s minutes of MVPA per day were significantly associated with greater self-efficacy (β = 1.34, *p* = 0.04) and number of family members reported in his network (β = 41.46, *p* = 0.004). While not statistically significant in multivariate analysis, the number of children reported by fathers was inversely associated with minutes of MVPA (β = −14.31, *p* = 0.06). Table 4 provides multivariate regression analysis of minutes of sedentary activity and MVPA per day.

## 4. Discussion

This paper aimed to better understand the social network factors attributing to the PA behaviors of Mexican-heritage fathers living in Texas *colonias* by using egocentric social network analysis. This preliminary study prioritized the specificity of this population over wide generalizability. Understanding the social environment and influence related to PA behavior among these fathers is all the more poignant considering Latinx adults carry a disproportionately high rate of obesity and obesity-related chronic disease such as diabetes and heart disease [8,9], and considering cultural norms impact the way Latinx individuals build and maintain their social networks [31]. Additionally, understanding the health implications of a father’s social network is supported by both family systems theory and the socio–ecological model [24,27].

Most of the fathers in this sample (65%) reported the maximum number of alters (*n* = 5). While limiting the nominations may have reduced the ability to capture weak ties, the goal was to obtain a closer look at the more salient connections regarding PA. In a similar study on Mexican-heritage adults, researchers asked participants to list “friends and family who have played a significant role in your life during the past year” with no limit on nominations [36]. This is a much broader network-generating question and hence obtained an average of 15.28 alters per network. Additionally, only 10.8% of total network members were said to encourage the participant to be active [36]. Fathers in this sample reported 91% of alters helped them be active. This perceived support is very promising as past researchers have found supportive network members significantly increased the intention to be active [36].

Fathers in this sample reported significantly more male alters than female alters and more family members than friends. Reporting more male alters would be a form of homophily or making a connection to someone because of a similarity [50]. Support from male peers and social interaction with other males was listed as a key component to motivate male adults to be active according to two qualitative studies [51,52]. Additionally, the propensity to report family members over friends would be consistent with collectivistic and familistic cultural values [33]. However, there was not a significant difference between the number of male children and female children reported by the fathers, meaning fathers are not more likely to be more active with one gender over another. This is important to note as PA behaviors of both male and female children have been associated with PA behaviors of their fathers [53,54]. Furthermore, some studies show girls can be stereotyped into less physically active extra-curricular activities and household roles, which can lead to less PA than their male counterparts [55], making it all the more important that they are active with their fathers.

A father’s minutes of MVPA per day were significantly associated with the number of family members reported in their networks. This focus on activity with family is in line with familism values [33] and supports other studies in which men are more active when their family members are more active [20]. Encouraging fathers to engage in physical activities involving the entire family may have ripple effects due to co-participation and concurrent play benefiting the health of the entire family [22]—they are more active, but so are their family members [19,56]. Furthermore, self-efficacy scores were positively related to MVPA in this sample. Consistent with existing evidence among other populations, self-efficacy is commonly associated with increased PA in adults and is often cited as essential to someone engaging in PA [40].

In multivariate analysis, a father’s minutes of sedentary activity per day were significantly associated with the number of friends he nominated, yet inversely associated with the number of children reported in his network. It is possible, fathers who listed more children may have spent more time caring for them, and in turn, playing and engaging with them. Encouragement from children was significantly associated with co-participation in PA among a sample of Mexican-heritage parent child dyads [57]. In general, these results implicate spending more family time or time playing with their children may help fathers be less sedentary. Providing opportunities for fathers to learn, connect, and play with their children in collaborative games may encourage this [58,59]. It could also be men who report more friends or coworkers in their network could either engage in less active hobbies with other friends (e.g., watching sports, playing poker) [60]. Subsequently, encouraging fathers to engage in active hobbies with friends may reduce sedentary time while increasing the stress relieving effects of leisure time PA.

The sample studied here had a relatively high mean minutes of MVPA per day. The mean measured MVPA per day in this sample of 73.3 min was substantially higher than national estimates of Latinx adult males [7]. In fact, all participants would have met and exceeded the recommendation of 150 min per week, and 89% (*n* = 42) would have surpassed the threshold (300 min per week) set for additional health benefits [2]. While it is impossible to say for certain with the data presented, but it is possible many of our fathers may work physically demanding jobs which inflates this number. Mexican-Americans are overrepresented in manual labor jobs such as farming and construction which are physically demanding and have led to negative mental and physical health consequences such as depression and overuse injuries [61,62]. In a meta-analysis, researchers found work-related PA was significantly associated with mental ill-health [63]. However, researchers also found there was an inverse reciprocal relationship between work-fatigue and leisure time PA in a longitudinal study [64]. In other words, adults who engaged in more leisure time PA felt less work-fatigue and vice versa. Encouraging fathers to engage in PA outside of work responsibilities (e.g., with family) may be beneficial for mental health and lessen work fatigue.

### 4.1. Implications

This is among the first articles to report on the social networks of Mexican-heritage fathers in relation to their PA. For this group of men, listing more family members in their network of people they are active with was significantly related with MVPA, while listing more friends was related with more sedentary behavior. These results support family-wide activity promotion and is evidence programs such as “Healthy Dads, Healthy Kids” could be especially useful in increasing activity for fathers and his family members [13,59,60]. Family level health promotion is important given notable health disparities faced by functionally rural border *colonias* communities, including higher rates of diabetes, mental health conditions, food insecurity, and obesity [65,66,67,68]. It also highlights the importance of creating healthy norms within family units, as PA tends to be most salient for men in this sample when their family members are also active (versus if their friends are active). These findings support future research taking a network perspective to understand PA, particularly by looking more closely at how family networks might promote healthy behavior choices [24].

### 4.2. Limitations

This study provides valuable specificity to the *colonia* community; however, this specificity has significant limitations regarding generalizability. Specificity was valued in this analysis to more accurately speak to the social influences exhibited on these fathers specifically within the community. The small sample size in this study limits the generalizability of findings, as well as the interpretation of findings. While some researchers have proposed general rules of thumb for 10–20 observations per independent variable or 50 observations total, the sample size in this study is at the bottom end of that recommendation and should be interpreted with caution [69,70]. Likewise, the factor analysis presented here was similarly underpowered, yet was calculated to add to the exploratory nature of this study. Future research should confirm these factor loadings and scale validity with a more robust sample. These results should be considered as pilot work in understanding the composition of Mexican-heritage fathers in a systematically underserved population. Similarly, the specific focus on this population residing in *colonias* limits generalizability but adds vital specificity to the social and cultural aspects of these networks. Furthermore, the added focus on functionally rural contexts would limit generalizability to urban areas yet strengthens the study as it relates to specificity of the population. As previously noted, the networks were limited to five participants which should be considered a limitation. It is possible this left out important connections; however, limits have been used in past studies in order to limit respondent burden and in an attempt to only elicit the most important connections [46]. Additionally, while it may be that fathers in this sample were accruing PA in work settings, we did not collect occupation information or require a daily log or recall to be able to delineate between leisure and work related PA. In future studies, delineating between work and leisure PA would be valuable.

## 5. Conclusions

Little is known about the social networks and social influences related to Latinx fathers residing in *colonias*, especially as they relate to PA and sedentary behavior. This study further contributes to the evidence by examining PA correlates of Latinx fathers from functionally rural *colonia* communities. Social influences related to Latinx father PA in this community provide tangible opportunities to promote familial PA through family-centered father-focused programing. Additionally, this study supports the use of family systems theory as social familial influences were associated with the PA behaviors of fathers in the study. Engaging the fathers in this community with their children in co-participation would be an ideal way to promote PA and reduce sedentary activity. Researchers and community health specialists seeking to promote PA and reduce sedentary behavior within this community would do well to involve familial and social influences when considering culturally and contextually tailoring health programing. Who these fathers report being active with, notably whether they are active with family rather than friends, is important in understanding PA engagement of Mexican-heritage adult men.

## Figures and Tables

**Table 1:** Confirmatory factor analysis factor loadings and scale means.

Item	Mean (SD)	Situational α = 0.78	Intrapersonal α = 0.94	Internal Feelings α = 0.70
How confident are you that you can be physically active for 30 min at least 5 times a week?	1.83 (0.48)	0.56		
How confident are you that you can be physically active when you are feeling tired?	1.17 (0.67)	0.59		
How confident are you that you can be physically active when you are feeling under pressure to get things done?	1.40 (0.68)	0.64		
How confident are you that you can be physically active when you are feeling down or depressed?	0.96 (0.83)	0.58		
How confident are you that you can be physically active when you have too much work to do?	1.34 (0.73)	0.55		
How confident are you that you can be physically active even when you have other more interesting things to do?	1.28 (0.74)	0.53		
How confident are you that you can be physically active when you don’t really feel like it?	0.83 (0.79)	0.65		
How confident are you that you can be physically active when you are traveling and away from home (e.g., traveling, visiting, on vacation)?	1.04 (0.86)	0.53		
How confident are you that you can be physically active even when your family or friends do not provide any kind of support?	1.28 (0.85)		0.60	
How confident are you that you can be physically active or play actively with your children?	1.89 (0.32)		0.68	
How confident are you that you can be physically active or play actively with your entire family?	1.91 (0.28)		0.68	
When it comes to being active, how confident are you that you have the ability to do it well?	1.79 (0.46)			0.50
How confident are you in being able to regularly exercise and be physically active?	1.57 (0.54)			0.74
How confident are you in being able to enjoy exercise and physical activity?	1.66 (0.60)			0.74

Note: SD—Standard deviation.

**Table 2:** Father network composition means and standard deviations.

Network Composition	Mean (SD)
Total network	4.36 (1.15)
Male Alters	2.81 (1.60)
Female Alters	1.57 (1.44)
Family	3.60 (1.64)
Children	2.09 (1.65)
Male Children	1.17 (1.07)
Female Children	0.91 (1.12)
Friend	0.77 (1.37)
Active Alters	3.74 (1.28)
Alters who help	4.00 (1.18)

**Table 3:** Pearson correlation coefficient for associations between network composition and minutes of Moderate-to-Vigorous Physical Activity (MVPA) and sedentary activity.

Covariate	MVPA	Sedentary Minutes
Total network	0.01	−0.14
Male Alters	−0.09	0.01
Female Alters	0.12	−0.11
Family	0.29 *	−0.08
Children	0.15	−0.31 *
Friend	−0.10	0.30 *
Active Alters	−0.08	−0.13
Alters who help	0.02	−0.20
Self-efficacy	0.29 *	0.07

* significant at *p* < 0.05.

**Table 4:** Multiple regression analysis results for sedentary activity and MVPA per day.

Covariate	MVPA (R^2^ = 0.25)		Sedentary Minutes (R^2^ = 0.19)	
	β	*p*-Value	β	*p*-Value
Family	41.46	0.004	−21.91	0.20
Children	−14.31	0.06	−55.86	0.01
Friend	−3.51	0.58	47.14	0.05
Self-efficacy	1.34	0.04	0.75	0.78

Note: MVPA—Moderate-to-Vigorous Physical Activity.
